# Investigation of Microbial Quality of Milk and Milk Products and Isolations of Some Major Bacteria in the Central and Northwestern Zones of Tigray, Ethiopia

**DOI:** 10.1155/vmi/9989527

**Published:** 2024-12-23

**Authors:** Dawit Gebremichael, Alem Tadesse, Fsahatsion Hailemariam, Birhane Hailay, Hagos Hadgu, Girmay Kalayu

**Affiliations:** ^1^Department of Animal Science, Aksum University, Shire Campus, Shire, Ethiopia; ^2^Department of Biology, Aksum University, Axum, Ethiopia

**Keywords:** cow milk, microbial load, milk quality

## Abstract

Safety and quality of milk and milk products are an increasing concern worldwide. Milk and milk products are major causes of milk-borne diseases due to contamination with microorganisms resulting from a lack of standard milk handling procedures and hygienic practices. Thus, the study aims to investigate the microbial quality and safety of cow milk and milk products and isolate some bacteria in Tigray. Questionnaires were conducted to assess milk handling procedures and hygienic practices. Samples were collected from different sampling points in the summer and winter seasons. Laboratory analyses were conducted using microbiological methods. SPSS version 20 was used to analyze the results. The overall mean total bacterial counts were 4.94, 6.02, 6.58, and 6.23 log10 CFU/mL for milk samples collected directly from the udder, milk container, cafeteria, and yogurt, respectively. Total bacterial counts exhibited statistically significant differences (*p* < 0.001) among different sampling points. The bacterial load in the winter season was significantly higher than in the summer season (*p* < 0.04). Highly significant differences in coliform counts were observed (*p* < 0.001) with mean values of 4.29, 5.49, 6.22, and 5.86 log10 CFU/mL for milk samples obtained directly from the udder, milk container, cafeteria, and yogurt, respectively. The averages of spore-forming and psychrotrophic counts were 4.13 and 5.40 log10 CFU/mL, respectively. *Escherichia coli*, Salmonella species, and *Staphylococcus aureus* exhibited significant variations at different sampling points. The isolation rates of Salmonella spp., *E. coli,* and *S. aureus* were 41.7%, 75%, and 95.8%, respectively. Total bacterial counts and psychrotrophic counts of the butter were 4.34 and 4.38 log10 CFU/g, respectively. Overall, the results indicate that milk and milk products had high levels of contamination because the bacterial loads were significantly higher than standard limits (5 log10 CFU/mL). Therefore, public education and awareness campaigns on good hygienic practices for dairy farmers and cafeteria owners are essential. Implementation of stringent food quality and safety standards, along with effective regulatory measures, is imperative to ensure safeguard consumer health.

## 1. Introduction

Milk and milk products are highly nutritious food [1, 2]. However, they are highly exposed to contaminations [3]. They are an ideal medium for the growth and proliferation of nonpathogenic and pathogenic bacteria, which can cause spoilage, infection, and intoxication [4, 5]. The quality and safety of milk and milk products depend on hygienic and sanitation practices, farm management, milk handling procedures and milking process, storage and methods of transportation, health conditions of animals, and water used to wash the milk utensils, udders, and teats, which are the major risk factors to prevent milk contaminations from microorganisms and protect the public and consumers from milk-borne diseases [6–10].

Milk and milk products are major potential causes of serious foodborne diseases in developing countries [6]. Raw milk and its derivatives can indeed harbor various pathogenic bacteria, including *Salmonella species, Escherichia coli*, *Staphylococcus aureus, Campylobacter* spp., and *Listeria monocytogenes*, which pose serious health risks when consumed by humans [11, 12]. Fecal contamination from sources such as water, food, and environmental factors can introduce coliforms, *E. coli*, and other enteric bacteria into milk and dairy products, further exacerbating the risk of foodborne illness [7, 12, 13]. Mastitis, an inflammation of the udder commonly caused by bacteria such as *S. aureus*, not only affects the health and welfare of dairy animals but also risks issues of the quality and safety of milk [6]. Moreover, nonpathogenic bacteria such as psychrotrophic organisms can contribute to milk spoilage, leading to economic losses for dairy producers and reduced milk quality for consumers [14, 15].

Addressing these concerns requires a multifaceted approach encompassing strict hygiene and sanitation practices, proper management of dairy animals, implementation of effective milking and milk handling protocols, and regular monitoring and testing for microbial contamination. Improving infrastructure for water quality management and investing in education and awareness programs for dairy farmers and consumers are also essential steps in mitigating the risks associated with milk-borne diseases [9, 16].

The situation described in the Tigray region of Ethiopia highlights significant challenges in ensuring the quality and safety of raw milk due to the predominance of informal marketing systems. The absence of government monitoring and regulation in the distribution of fresh raw milk poses a substantial risk to public health [12]. The lack of standardized hygienic parameters, enforcement mechanisms, and regulatory frameworks further compounds the problem, making it difficult to maintain consistent milk quality standards. Inadequate transportation methods and the widespread practice of door-to-door raw milk distribution only serve to exacerbate the prevalence of microbial contamination, increasing the likelihood of foodborne illnesses among consumers [17–19]. Addressing these issues requires coordinated efforts from government authorities, dairy industry stakeholders, and local communities. Implementing regulations and enforcement mechanisms to ensure compliance with hygiene standards and quality control measures is crucial. Additionally, investments in infrastructure improvements, training programs for dairy farmers and vendors, and public awareness campaigns can help promote safer milk production and distribution practices [20].

Conducting research to investigate the microbial quality of milk and milk products, as well as isolating selected bacteria in the central and northwestern zones of Tigray, is a crucial step toward addressing the information gaps and improving milk safety practices in the region. By undertaking such research, valuable data can be generated to assess the microbial loads present in milk and dairy products, identify specific pathogens or contaminants, and evaluate the effectiveness of current milk handling practices. This information is essential for understanding the extent of the problem and formulating evidence-based strategies to mitigate risks and ensure the safety of milk for consumers. Furthermore, the findings of this research can provide valuable insights to the government of the Tigray regional state and other stakeholders, helping them make informed decisions regarding policy development, regulatory measures, and public awareness campaigns aimed at promoting safer milk production, handling, and consumption practices. Collaboration with academic institutions such as Axum University demonstrates a commitment to leveraging scientific expertise and research capabilities to address pressing public health concerns and improve the overall quality of life for residents in the Tigray region. By prioritizing research on milk safety and microbial quality, the government and stakeholders in Tigray can take proactive steps toward enhancing food safety standards, protecting public health, and fostering sustainable development in the region. Therefore, the research aims to investigate the microbial quality of milk and milk products and isolations of some major bacteria in the central and northwestern zones of Tigray.

## 2. Methods and Materials

### 2.1. Description of the Study Area

**Figure 1:**
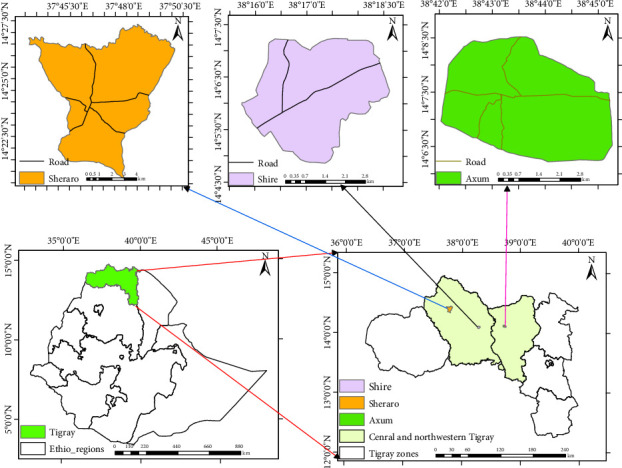
Map of the study area.

The study was conducted in the central and northwestern zones of the Tigray region, focusing on three specific towns: Axum, Shire, and Sheraro (Figure 1). Axum is located in the central zone of Tigray about 963 km away to the north from Addis Ababa, Ethiopia, and 202 km from Mekelle, the capital city of the Tigray region, at an average elevation of 2130 m above sea level. The geographical coordinates are 14° 7′ 8″ N and 38° 43′ 46″ E. The average daily temperature of the town is 12.8°C in the wet season and 28.8°C in the dry season with an average annual rainfall of 652 mm and an average relative humidity of 32%. Shire is located in the northwest zone of Tigray 63 km away to the west from Axum at 14.1057 N and 38.2849 E geographical coordinates with an average elevation of 1953 m above sea level. This town has an average annual rainfall of 905 mm with an average daily temperature of 18°C in the wet season and 34.6°C in the dry season. The average relative humidity is 27%. Sheraro town is also found in the northwest zone of Tigray located at a distance of 95 km from Shire to the west in geographical coordinates of 14.3970 N and 37.7743 E with an altitude ranging from 1040 to 1246 m above sea level. The average annual rainfall of the area ranges from 400 to 500 mm, and the average temperature is 33°C in the wet season and 40°C in the dry season with an average relative humidity of 24% [21]. These descriptions provide a comprehensive overview of the geographical locations, elevations, climates, and other relevant environmental factors of the towns where the study was conducted, offering insights into the context in which the research took place.

### 2.2. Study Population

The study population consisted of dairy cows and dairy farms in the selected towns within the central and northwestern zones of the Tigray region. A total of 1670 dairy cows and 344 dairy farms were found in Axum, while 1530 dairy cows and 332 dairy farms were found in Shire. In Sheraro, a total number of dairy cows and dairy farms were 1345 and 303, respectively. In total, the study encompassed 4545 dairy cows and 979 dairy farms across the three towns. This population served as the basis for data collection and analysis regarding milk production, handling practices, and microbial quality assessments. By including a wide range of dairy farms and cows, the study aimed to provide comprehensive insights into the milk and milk product supply chain within the region.

### 2.3. Study Design and Sampling Methods

The study employed a cross-sectional design to analyze the microbial quality and safety of cow milk and milk products over a period spanning from December 2018 to July 2020. Three towns were purposively selected from the central and northwestern zones of Tigray based on criteria such as milk production potential, accessibility, and population density. These towns were chosen to represent different agroecology zones: Axum (highland), Shire (midland), and Sheraro (lowland). Milk and milk product samples were collected from critical points along the milk value chain, including the udder, milk containers, and cafeterias. All samples were obtained from crossed dairy cows (HF with local zebu cattle). Axum, Shire, and Sheraro have 5, 5, and 4 “*Kebeles or small administrative unit*,” respectively. However, 3, 4, and 4 “*Kebeles*” were obtained from Axum, Shire, and Sheraro, respectively, based on the accessibility and potential of dairy farms. In Axum, a total of 35 farms and 150 lactating cows were included in the study with a range of three to six cows per farm. A total of 35 farms and 140 lactating cows were also included with a range of three to five cows per farm. Similarly, 35 farms and 133 dairy lactating cows were included with a range of two to five cows per farm. Milk samples collected from a single farm were pooled together and considered as one sample. A total of 105 milk samples were collected from the udder and milk containers/buckets at the farm level using a simple random sampling method during the wet (summer) season. This included 35 samples or farms from each town. Additionally, 105 milk samples (35 from each town) and 105 yogurt samples (35 from each town) were collected from cafeterias during the wet (summer) season. Yogurt (also known as *ergo*) is naturally fermented milk through the application of traditional fermentation methods, and also, several spices and the process of smoking with herbs are used to improve flavor, palatability, and quality. Spices and herbs are also used to inhibit microbial growth. In addition, 30 butter samples (10 from each town) were collected from the open market during the dry (winter) season. Raw milk and yogurt samples were also collected during the dry (winter) season to examine microbial loads across different seasons, while butter samples were only examined during the dry season.

### 2.4. Questionnaire Survey

The questionnaire survey conducted as part of the study aimed to comprehensively assess milk handling and hygienic practices among dairy cow owners and farm attendants in the three study areas. A total of 105 semistructured questionnaires were conducted, with 35 questionnaires distributed to 35 dairy farm owners in each of the three towns: Axum, Shire, and Sheraro. The questionnaires were designed to gather detailed information on milk handling and hygienic practices from dairy cow owners and farm attendants. Contents of the surveys were focused on hygiene procedures and sanitary conditions of the barn and milking environment, waste management, the availability and sources of water for milking and cleaning purposes, inquiries about hand hygiene, udder cleaning, and overall cleanliness during the milking process, container types, cleaning frequency, milk transportation systems, and milk storage conditions. In addition to the questionnaire survey, observations were conducted on farm management practices and hygienic procedures to complement the self-reported data.

### 2.5. Sample Collection Methods

Laboratory-based investigation of this research project was targeted at determining the microbiological quality and safety of raw milk, yogurt, and butter samples. Raw milk samples were collected from the udder from each quarter and milking containers/buckets from 105 dairy farms (35 from each town) immediately after milking for analysis. Raw milk and yogurt samples were also collected from the cafeterias, while butter samples were collected from open markets. First, milk samples were collected directly from the udders into sterilized bottles. Then, after collecting samples from the udder, farmers milked their cows into one milk container. After that, milk samples were collected from the farmer's milk container into a sterilized bottle. Both milk samples collected directly from the udder and milking container were at the dairy farm level. Besides, milk samples collected from the cafeterias were pooled in a sterile bottle. A similar procedure was performed for yogurt and butter samples collected from the cafeterias and open markets. Two hundred and fifty milliliters of milk samples collected from the udder, milk container/buckets, and cafeterias was transferred into a sterile screw-capped bottle. In addition to the raw milk analysis, 250 mL of yogurt and 250 g of butter samples were also obtained from the cafeteria and open market, respectively. All samples were labeled and put in the icebox (4°C) to restrict microbial multiplication and transported as early as possible to the laboratory of Axum University to analyze the microbial quality. Samples arrived at the laboratory site within 12 h.

### 2.6. Microbiological Test

#### 2.6.1. Total Bacterial Count (TBC)

Standard guidelines for the preparation of milk and milk product samples were used based on the methods of ISO 8261:2001 [22]. One milliliter of milk and yogurt and 1 g of butter samples were added into a sterile test tube having 9 mL of peptone water. Medium (plate count agar—Oxoid 325) was prepared, and 15 mL of the molten agar was poured into sterile petri dishes in duplicate according to the manufacturer's instructions. From each dilution rate (test tube), 1 mL was transferred to two petri dishes from 10^−4^ and 1 mL to two petri dishes from 10^−5^ dilution rates. The bacterial suspension was mixed, and the agar was set aside to solidify, and then, it was incubated at 30°^C^ for 72 h. The numbers of colony-forming units (CFUs) were counted using the Quebec colony counter. The results were calculated using ISO 8261:2001 [22].

#### 2.6.2. Total Coliform Count (TCC)

A general guideline on quality assurance was used for the preparation of culture media based on ISO/TS 11133-1:2009 procedures [23]. One milliliter of milk and yogurt and 1 g of butter samples were added into a sterile test tube having 9 mL of peptone water. Then, 1 mL was transferred to 15–20 mL Violet Red Bile Agar (VRBA) solution. After thorough mixing, the plated sample was permitted to solidify. Then, petri dishes were incubated at 32°C for 24 h [23]. Only dark red colonies were counted. ISO/TS 11133-1:2009 procedures were used to calculate the results (Figure S1).

#### 2.6.3. Mesophilic Spore Form Count

Mesophilic spore form counts were enumerated using methods described by Kent et al. [24]. The raw milk samples collected from the udder, milk container at the farm level, and cafeterias were heated at 80°C for 10 min to destroy vegetative cells. After being cooled in an ice bath, the sample will be immediately plated on plate count agar and incubated at 32°C for 48 h. All small and large white colonies were counted, and the same formula was used to determine TBC [22].

#### 2.6.4. Psychrotrophic Bacterial Count

Similar to TBCs, a standard guideline was used for the preparation of milk and milk product samples based on the methods described by ISO 8261:2001 [22]. Psychrotrophic colony count was determined using plate count agar (DIFCO) after incubation at 7°C for 10 days. All small and large white colonies were counted. The total psychrotrophic count/mL or gm of the examined sample was calculated using the formula of ISO 8261:2001 [22].

#### 2.6.5. Enumeration and Isolation of Some Selected Bacteria

##### 2.6.5.1. *S. aureus*

Milk and milk product samples were used for enumeration of *S. aureus*. The serial dilution method for a total count on plate count agar was also followed on mannitol salt agar (Oxoid, England) for presumptive *S*. *aureus* load count. The presumptively identified *S*. *aureus* from mannitol salt agar was subcultured to the nutrient agar plate. Inoculated plates were incubated at 37°C for 48 h, and *S*. *aureus* counts per milliliter or gram of tested product were calculated and recorded. Those forming clumping of cocci were obtained as positive. Typical *S. aureus* colonies, which appeared as golden yellow, smooth, circular, and convex, were counted. Further confirmation of *S. aureus* colony was made using biochemical tests, which included methyl red test, catalase test, coagulase test, mannitol fermentation test, and Voges–Proskauer test using the methods of Quinn et al. [25].

##### 2.6.5.2. *E. coli*

For the enumeration of *E. coli*, 25 mL of milk, 25 mL of yogurt, and 25 g of butter samples were blended separately in 225 mL of lactose broth (Oxoid, England), and serial dilutions were made by adding 1 mL of the diluted sample suspension into each of the serially arranged test tubes containing each 9 mL of sterile saline solution. The eosin methylene blue (EMB) agar plates were incubated at 37°C for 24 h. Finally, blue–black with metallic green sheen colonies were counted (Figure S2). The pure colonies were subcultured on blood agar (Oxoid Ltd, Germany) and nutrient agar (Oxoid Ltd, England), and biochemical tests including the indole, methyl red, Voges–Proskauer, and citrate (IMViC) test were carried out for further confirmation using procedures of Quinn et al. [26].

##### 2.6.5.3. *Salmonella* spp.

25 mL of milk and 25 mL of yogurt samples were added separately into 225 mL of buffered peptone water at 35°C for 24 h. Similarly, 25 g of butter was added to 225 mL of buffer peptone water. From this, a 10 mL sample was inoculated into 90 mL selenite cystine broth (Oxoid, England) for selective enrichment and the suspension was incubated at 35°C for 24 h. After incubation at 35°C for 24 h, colorless colonies with black centers were recorded as positive (Figure S3). *Salmonella* colonies, having a slightly transparent zone of a reddish color and a black center, were subcultured on blood agar (Oxoid Ltd, Germany) and nutrient agar (Oxoid Ltd, England) [26]. Biochemical tests such as methyl red, urease, indole, and citrate tests were conducted for confirmation (Figure S4).

### 2.7. Data Analysis

The data generated from the survey and laboratories were entered into MS Excel spreadsheets and analyzed using SPSS version 20. The survey data were described using descriptive and inferential statistics such as means, frequency distribution, and percentage. Microbiological counts were transformed into the logarithmic scale (log10 CFU/mL or CFU/g). The one-way ANOVA test and univariate analysis were used to test for statistically significant differences between seasons, towns, and different sampling points. The significance level was set at *p* < 0.05, indicating that results with *p* values less than 0.05 were considered statistically significant.

## 3. Results

### 3.1. Questionnaire Survey

Among 105 interviewed, 98.1% and 90.5% of them washed their hands and the udders of the cows before milking, respectively. Around 81% of dairy producers used cold water with soap to wash their hands. Only 17.9% of dairy farmers used warm water to wash the udders of the cows. Only 24.8% of dairy farmers used a towel to dry the udder after washing, while 41.9% of dairy farmers indicated that they did not wear any uniform during milking (Table 1).

Availability of clean water is one of the challenges in all districts of the study. Around 96.2% of the dairy farms had a scarcity of clean water. Among the total of 105 respondents, only 30 (28.6%) of them used tap water/clean water, while 66 (62.9%) and 9 (8.6%) of the dairy farms used wells and rivers, respectively. Only 4.8% and 3.8% of the dairy farmers used stainless steel containers for milking and transportation, respectively. After milking and bulking at the herd level, all milk was found to be transported through 37.1% on foot, 46.7% by cycle, and 12.4% by Bajaj. Among 105 respondents, only 8.6% of the respondents indicated that they have awareness about sources of milk contamination (Table 2).

### 3.2. TBC

Raw milk samples were collected directly from the udder, milking buckets, and cafeterias to determine TBCs in three different districts. Yogurts were also collected from cafeterias. The overall mean TBCs of milk samples collected directly from the udder, bucket, and cafeteria and yogurt samples collected from the cafeteria were 4.94, 6.02, 6.58, and 6.23 log10 CFU/mL, respectively. There were statistically significant differences (*p* < 0.001) among samples obtained from the udder, bucket, cafeteria, and yogurt. The milk sample collected from the cafeteria was the most contaminated/bacterial loaded, whereas the milk sample from the udder was the least contaminated/bacterial loaded. The maximum recommended value of the microbiological standards of the European Union (EU) is 5.00 log10 CFU/mL and 2.00 log10 CFU/mL for TBC and coliform count (CC), respectively [22, 24]. There was a statistically significant difference (*p* < 0.04) in mean TBC between the winter and summer seasons. The winter season exhibited higher bacterial loads than the summer season, with mean values of 6.25 log10 CFU/mL and 5.64 log10 CFU/mL, respectively. Statistically significant variation was not observed in milk samples collected from the three districts (Table 3).

### 3.3. CC

The mean value of CCs of milk samples obtained from the udder, milk containers/buckets, cafeterias, and yogurt samples obtained from cafeterias were 4.29, 5.49, 6.22, and 5.86 log10 CFU/mL, respectively. The mean value of CC exhibited a highly significant difference (*p* < 0.001) with the highest bacterial load in a sample obtained from the cafeteria. The season did not show a statistically significant difference (*p* > 0.05) in TCCs. This suggests that CCs remained consistent across seasons. Generally, bacterial counts appeared to be quite consistent across all districts. However, lower CCs were observed in samples collected directly from the udder compared with other sources (Table 4).

### 3.4. Spore-Forming Count

The overall average total spore-forming counts were 1.93, 4.89, and 5.57 log10 CFU/mL) for milk samples collected directly from the udder, milk containers/buckets, and cafeteria, respectively. There was a significant difference (*p* < 0.001) in spore-forming counts between milk samples collected from cafeterias compared with those collected directly from the udder. Milk from milking containers/buckets also had significantly higher spore-forming counts than milk from the udder. Season and districts did not show significant differences (*p* > 0.05) in spore-forming counts. This suggests that spore-forming counts were consistent across seasons and towns (Table 5).

### 3.5. Psychrotrophic Count

The results in Table 6 showed that the mean counts of the total psychrotrophic bacteria were 5.40 log10 CFU/mL. There was no significant difference observed among the different sampling milk sources in terms of psychrotrophic counts. This suggests that psychrotrophic bacterial levels were consistent across milk sources. The average total psychrotrophic counts of cow's raw milk and yogurt in the summer and winter seasons were 5.94 log10 CFU/mL and 4.93 log10 CFU/mL, respectively. A highly significant variation (*p* < 0.001) was observed between the summer and winter seasons in terms of the total psychrotrophic count. The winter season exhibited lower psychrotrophic counts than the summer season. The mean value of the total psychrotrophic count was slightly significantly higher in Axum (5.94 log10 CFU/mL) than in Sheraro (5.07 log10 CFU/mL).

### 3.6. Enumeration of Some Major Bacterial Species

*E. coli*, *Salmonella* species, and *S. aureus* were significant variations along with different sampling points (*p* < 0.05). The microbial contaminations were increased in milk samples collected from the udder to cafeterias (Table 7). All of them did not observe significant differences among different districts. Only *S. aureus* showed strong significant variations (*p* < 0.008) between the two seasons. The summer season had higher *S. aureus* counts than the winter season (Table 8).

### 3.7. Determination of Bacterial Load of Butter

**Figure 2:**
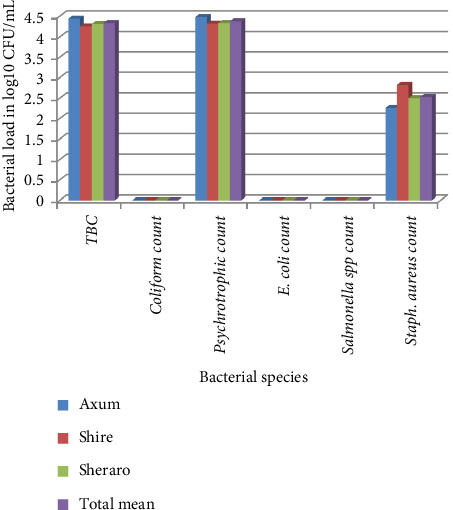
Determination of bacterial load of butter in log10 CFU/g.

The TBC, psychrotrophic count, and *S. aureus* count of butter were 4.34, 4.38, and 2.53 log10 CFU/g, respectively. The TBC and psychrotrophic count were slightly higher than in Shire and Sheraro, but the *S. aureus* count was lower in Axum than in Shire and Sheraro. There was no contamination of the butter with the coliform bacteria, *E. coli,* and *Salmonella* spp. (Figure 2).

### 3.8. Isolation of Major Bacterial Species

**Figure 3:**
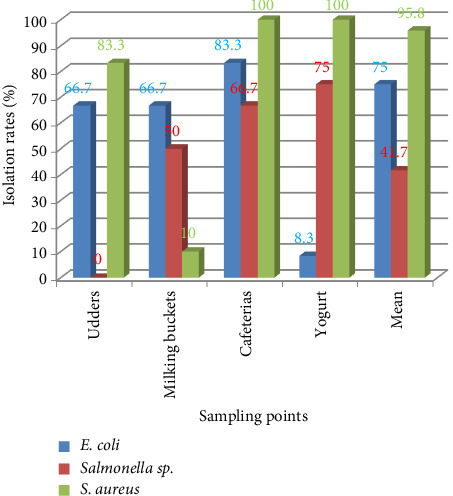
Major bacterial species isolated from milk and yogurt.

The isolation rates of *Salmonella* spp., *E. coli,* and *S. aureus* were 41.7%, 75%, and 95.8%, respectively (Figure 3).

## 4. Discussion

Raw milk and milk products are extremely perishable and spoil very quickly. Milk and its products are a perfect food for microbial growth [6, 20, 27]. In Ethiopia, milk and its products often occur in unhygienic situations, and consuming raw milk is common [28]. The standard EU microbiological limits of cow milk for a TBC to accept for human consumption were 5.00 log10 CFU/mL [22, 24]. The mean of TBC of milk collected from buckets and cafeteria and yogurt was higher than the maximum recommended value of the microbiological standards of the EU (> 5 log10 CFU/mL). The average TBC was 5.94 log10 CFU/mL in the study area. However, the bacterial load of milk obtained from the udder was at an acceptable level (< 5 log10 CFU/mL). The prevalence of high TBCs exceeding the EU standards in milk collected from buckets, cafeterias, and yogurt underscores the significant risk posed to consumers. This contamination can be attributed to various factors such as inadequate hygiene practices during milking, poor environmental sanitation, and improper storage conditions. TBC of this result is similar to the findings of Tegegne and Tesfaye [9] with a bacterial load of 4.59 log10 CFU/mL but lower than the findings of Mohamed et al. [15], Worku et al. [29], Yilma [30], and Amakelew et al. [31] with a bacterial load of 6.78, 7.59, 8.87, and 7.25 log10 CFU/mL, respectively. The TBC is the most common technique used for assessing the quality of raw milk [32]. A high amount of TBC in the raw milk and milk products indicated unhygienic and unsanitary conditions of milk handling and milking practices [12], low environmental sanitation and poor milking practices [20], inappropriate washing of the udder [6], and unsuitable storage conditions of milk [2]. In addition, higher microbial contaminations of TBC than the EU standards might be because most of the dairy cow owners only used cold water to clean milk equipment.

TCCs greater than the standard EU (2.00 log10 CFU/mL) microbiological limits reveal sanitation-related problems in dairy farms [6, 23]. The CC in the study was 5.47 log10 CFU/mL. The average CC is similar to the findings of Amakelew et al. [31] and Naing et al. [32] in that the CC was 5.47 and 5.2 log10 CFU/mL, respectively. However, the results were higher than the results of 3.91, 4.49, 4.58, 4.18, and 4.03 log10 CFU/mL reported by Mohamed et al. [15], Asaminew and Eyassu [33], Zelalem [34], Abebe et al. [35], and Abera et al. [36], respectively. The elevation of CC in milk indicates low hygienic procedures and sanitation practices in dairy farms [32]. The results revealed that 62.9% of respondents stated that their sources of water were open wells, which were used for washing milking materials, udder, hands, etc. Given that water from open wells may not meet safety standards for cleanliness, its use in dairy farm operations poses a considerable threat to milk hygiene and safety [37–39].

The statistical analysis reveals significant differences in both TBCs and CC among samples collected from different sources, with contamination levels escalating from the udder to the cafeteria. This finding underscores the multifaceted nature of milk handling and distribution processes, which involve multiple factors and increase the likelihood of microbial contamination from various sources [39]. The current study revealed that the mean TBCs were higher than the EU standard (5 log10 CFU/mL) except milk samples collected directly from the udders. The discussion highlights seasonal variations in bacterial contamination levels, with winter showing a significantly higher bacterial load than summer. Similarly, seasonal variations in bacterial contamination levels were reported by Nateghi et al. [40].

The current study revealed that the loads of coliform bacteria were slightly higher in the summer season. Coliforms are the main cause of subclinical mastitis, and CCs in milk were significantly higher during the wet season than the dry season. The current results were similar to Gouranga et al. [41] who found that the highest occurrence of TBCs (5.64 log10 CFU/mL) was during the winter season, whereas the lowest occurrence of TBCs (3.78 log10 CFU/mL) was during the summer season. The TCC of raw cow milk samples collected during the wet season was higher than in the dry season [42]. Gillespie et al. [43] also reported that CC was significantly higher during the summer season than the winter season. The variation may depend on the temperature and rain status. In Ethiopia, the temperature increases in the winter season than in the summer season, but this may not be true for other countries. The average temperatures of the three study areas are 21.27°C and 34.47°C in the wet season and dry season, respectively. TBC reflects a more active metabolism during the dry season. Besides, the questionnaire survey revealed that scarcity and unavailability of pure water were the main challenges in the dairy farms of the study area in the dry season. However, a supply of pure and sufficient water was available in the summer season in the study area. This is one of the possible reasons to reduce microbial contamination in the summer season.

Generally, all milk samples in this study had higher spore-forming loads than the recommended level of EU [9, 22]. The results generally showed significant differences (*p* < 0.001) in spore-forming bacterial counts across milk sources. The average mean count of spore-forming bacteria in raw milk samples was 4.13 log10 CFU/mL, which was lower than 5.29 log10 CFU/mL reported by Debela [44]. The relatively higher spore-forming bacterial count in milk samples obtained from the cafeterias may be due to poor handling practices at the vending sites [45–48].

The discussion highlights the findings regarding psychrotrophic bacterial counts in raw milk and yogurt samples, emphasizing their significance in assessing milk quality and safety. Psychrotrophic bacteria are a group of microorganisms capable of growing at refrigeration temperatures, posing a risk to the quality and shelf life of milk and dairy products. The mean psychrotrophic counts reported in the study were 5.40 log10 CFU/mL in raw milk and 4.70 log10 CFU/mL in yogurt, exceeding the recommended limit of 5.00 log10 CFU/mL for total microorganisms in milk products [24]. The result was higher than the result of Özdemir et al. [49], but it is lower than the report of Amer et al. [50] in which the bacterial loads were 2.39 and 6.50 log10 CFU/mL, respectively. The average total psychrotrophic count of cow's raw milk and yogurt exhibited highly significant variation between the summer and winter seasons. This is due to the variation of temperature in the study area. The average temperature of the winter season is higher than the summer season in the central and northwestern zones of Tigray. The average temperature is 21.27°C and 34.47°C in the winter and summer seasons, respectively, in the study areas. The summer season is suitable for the multiplication of psychrotrophic bacteria than the winter season. The mean value of total psychrotrophic count was slightly significant in Axum than in Sheraro. This variation can also be due to the difference in agroecology of the study area. Axum is a highland with an elevation of 2130 m above sea level, while Sheraro is a lowland with an elevation of 1246 m above sea level. Generally, Axum is colder than Sheraro and favors the multiplication of cold-loving bacteria. Thus, the psychrotrophic bacteria had higher loads in Axum than in Sheraro and Shire. Psychrotrophic bacteria cause spoilage of milk, and its products result in low quality of milk and high economic losses [50, 51].

The overall prevalence of *E. coli, Salmonella* sp., and *S. aureus* in different milk samples was 75%, 41.67%, and 95.83%, respectively. The prevalence of *S. aureus* was strongly higher than in the study conducted by Regasa et al. [2], Ayele et al. [4], Reta et al. [7], Tegegne and Tesfaye [9], and Tigabu et al. [52] where the prevalence of *S. aureus* in different milk samples was 16.6%, 19.6%, 24.2%, 25%, and 24%, respectively. Higher isolation frequencies of *E. coli* were observed in the current study as compared to the results of Reta et al. [7] and Faisal and Ahmed [53] with the prevalence of 58% and 17.6%, respectively, in Ethiopia, and Addo et al. [54] with the prevalence of 11.2% in Ghana. Similarly, the prevalence of *Salmonella* spp. was higher than that of Akoachere et al. [55] in Cameroon with the prevalence of 27%.

The microbial loads of *E. coli, Salmonella* sp., and *S. aureus* were significantly higher in milk collected from cafeterias and milk containers than in milk collected directly from the udder (*p* < 0.05). Of particular concern was the significantly higher microbial load observed in milk collected from cafeterias and milk containers than in milk collected directly from the udder. The use of nonstainless steel milk containers and inadequate cleaning practices likely contributed to the increased bacterial load in these samples. Additionally, the study noted that traditional methods of milk distribution and transportation may further exacerbate microbial contamination, highlighting the need for improved hygiene practices throughout the milk production and distribution chain. In addition, regular opening of containers of milk in the cafeteria users is common and may result in increasing bacterial load [42]. According to Mhone et al. [56], milk can be contaminated with *E. coli* and *Salmonella* spp. from polluted water sources and when farms have poor drainage systems. Most of the dairy farms explained that the drainage system is one of the most challenges, especially in Shire town.

Mastitis, an inflammatory condition of the udder, directly impacts milk quality and safety, posing significant challenges to dairy production [57, 58]. Bacterial pathogens, including *E. coli* and *S. aureus*, are common causes of mastitis, contributing to decreased milk quality and increased risk of contamination [59]. The study observed a significantly higher prevalence of *S. aureus* in milk samples collected during the summer season than in the winter season (*p* < 0.008). This finding aligns with the understanding that mastitis incidence tends to be higher in warmer months. The increased prevalence of mastitis during the summer season may be attributed to various factors, including environmental conditions conducive to bacterial growth and increased milk production.

Research by Sinha et al. [60] and Suresh et al. [61] supports this observation, indicating a higher prevalence of mastitis during the summer season than in the winter season. The rainy season, characterized by higher humidity and precipitation, may create favorable conditions for bacterial proliferation, contributing to increased mastitis incidence. The greater occurrence of mastitis during the summer season poses significant challenges to dairy farmers, as it can lead to decreased milk quality, reduced milk production, and economic losses. Effective mastitis management strategies, including proper hygiene practices, regular udder health monitoring, and prompt treatment of infected animals, are essential for minimizing the impact of mastitis on milk quality and safety [59].

The study found that the TBC, psychrotrophic count, and *S. aureus* count of butter were within acceptable limits, indicating good microbial quality. Notably, there were no detectable counts of coliforms, *E. coli*, or *Salmonella* spp. in the butter samples, further confirming its safety and quality. The microbial quality of yogurt and butter was generally good as compared to international standards. Yogurt (*ergo*), spontaneously fermented milk, is the major raw material for the manufacturing of different Ethiopian traditional dairy products. In Ethiopia, fermented dairy products are traditionally produced by leaving fresh milk to spontaneously ferment for two or more days in presmoked traditional milk containers. Smoking milk containers using different plant species is a traditional practice in the manufacturing of Ethiopian dairy products [62]. The smoke enhances the taste and aroma of dairy products and helps in decontaminating the container due to its antimicrobial activity by reducing spoilage microorganisms and thereby extending the shelf life of the product [63]. According to Admasu et al. [64], TBCs, CCs, and *E. coli* counts of yogurt (*ergo*) of Ethiopia exhibited lower loads than the modernly prepared yogurt. Generally, the microbiological analyses indicate that ergo samples were safe for consumption.

Butter is also prepared and fermented traditionally as yogurt in Ethiopia. Several factors likely contribute to the favorable microbial quality of butter in the study area. Traditional butter processing methods, such as boiling and the addition of various spices, may have antimicrobial properties that help reduce bacterial contamination. Additionally, the practice of smoking butter containers with different woods and leaves containing microbial inhibitors during processing could contribute to lowering the bacterial load in butter [59].

## 5. Conclusions

The conclusions drawn from the study emphasize the critical need for immediate action to address the significant microbial contamination observed in milk and milk products. The study found alarming levels of contamination, including TBC, CC, spore-forming count, and presence of pathogenic bacteria such as *E. coli*, *Salmonella species*, and *S. aureus.* These findings indicated a serious health risk to consumers. Poor milk handling and hygienic practices, inadequate water supply, deficient transportation systems, lack of refrigeration facilities, and insufficient awareness of contamination sources were identified as key contributing factors to microbial contamination in milk and milk products. Overall, this study provides valuable insights into the microbial quality and safety challenges facing the dairy industry in Tigray and emphasizes the importance of concerted efforts to address these issues and safeguard public health. Therefore, immediate interventions are needed to address the identified shortcomings in milk production, handling, and distribution processes. Improving access to clean water, enhancing transportation systems, and implementing proper storage and refrigeration facilities are crucial steps to mitigate contamination risks. Public education and awareness campaigns on good hygienic and sanitary practices for dairy farmers and cafeteria owners are essential. Increasing knowledge about food safety standards, laws, and regulations can help reduce the incidence of foodborne illnesses and protect public health. Implementation of stringent food quality and safety standards, along with effective regulatory measures, is imperative to ensure compliance with microbiological standards and safeguard consumer health [65].

## Figures and Tables

**Table 1:** Milk hygienic practices.

Variables	Alternatives	No.	%
Washing your hands before you start milking	Yes	103	98.1
	No	2	1.9

Using of cleaning agent	Cold water and soap	85	81
	Hot water and soap	8	7.6
	Only cold water	12	11.4
	Only hot water		

Washing of the milking material before milking	Yes	104	99.0
	No	1	1.0

If yes, what do you use for washing the milking material?	Using water only	8	7.7
	Using water and detergent	89	85.6
	Others	7	6.7

Udder washing before milking	Yes	95	90.5
	No	10	9.5

Cleaning agent for udder	Cold water	78	82.1
	Hot water	17	17.9

Using any towel for udder drying before milking	Yes	79	75.2
	No	26	24.8

Wearing of uniform milking cloth during milking	Yes	61	58.1
	No	44	41.9

**Table 2:** Milk handling practices and sources of contaminations.

Variables	Alternatives	No.	%
Availability of clean water	Scarcity	101	96.2
	Adequate	4	3.8

Sources of water for hand, milk containers, and udder washing	Well	66	62.9
	River	9	8.57
	Tap water	30	28.6

Types of materials used for milking, milk storage, and transportation	Plastic	100	95.2
	Stainless steel	5	4.8

Means of milk transportation	On foot	39	37.1
	By cycle	49	46.7
	Bajaj	13	12.4
	Others	4	3.8

Time taken to reach the milk from the farm to the consumer	Less than 30 min	2	1.9
	30 min to 1 h	76	72.4
	Greater than 1 h	27	25.7

Any awareness about sources of milk contamination	Yes	9	8.6
	No	96	91.4

Knowledge/awareness of milk-borne zoonotic diseases	Yes	44	41.9
	No	61	58.1

Do you know about mastitis?	Yes	88	83.8
	No	17	16.2

If yes, do you milk mastitis cows?	Yes	60	68.2
	No	28	31.8

**Table 3:** Total bacterial count at different sampling points in log10 CFU/mL.

	Sampling points	Mean	SE	Min.	Max.	95% confidence interval for the mean		*p* value
						Lower bound	Upper bound	
Sampling points	Udder	4.94	0.29	4.13	5.82	4.56	5.32	< 0.001
	Milk from container/bucket	6.02	0.18	5.47	6.47	5.65	6.40	
	Cafeteria	6.58	0.07	6.42	6.74	6.20	6.96	
	Yogurt	6.23	0.10	5.93	6.57	5.85	6.61	
	Total	5.94	0.15	4.13	6.74	5.75	6.13	

Season	Summer	5.64	0.24	4.13	6.43	5.22	6.06	0.04
	Winter	6.25	0.16	4.86	6.74	5.83	6.66	
	Total	5.94	0.15	4.13	6.74	5.65	6.24	

Districts	Axum	5.862	0.3150	4.26	6.72	5.29	6.44	0.90
	Shire	5.929	0.30	4.13	6.73	5.35	6.50	
	Sheraro	6.035	0.20	4.86	6.74	5.46	6.61	
	Total	5.94	0.15	4.13	6.74	5.61	6.27	

Abbreviations: Max., maximum; Min., minimum; SE, standard error of mean.

**Table 4:** Total coliform count at different sampling points in log10 CFU/mL.

	Sampling points	Mean	SE	Min.	Max.	95% confidence interval for mean		*p* value
						Lower bound	Upper bound	
Sampling points	Udder	4.29	0.14	3.96	4.83	3.95	4.63	< 0.001
	Milk container/bucket	5.49	0.15	4.91	5.93	5.15	5.84	
	Cafeteria	6.22	0.17	5.44	6.61	5.88	6.57	
	Yogurt	5.86	0.20	5.21	6.44	5.51	6.20	
	Total	5.47	0.17	3.96	6.61	5.29	5.64	

Season	Summer	5.56	0.23	4.13	6.61	5.06	6.07	0.05
	Winter	5.37	0.26	3.96	6.55	4.86	5.87	
	Total	5.47	0.17	3.96	6.61	5.11	5.82	

Districts	Axum	5.45	0.29	4.13	6.55	4.81	6.09	0.94
	Shire	5.40	0.30	3.96	6.61	4.76	6.04	
	Sheraro	5.55	0.33	4.13	6.44	4.91	6.18	
	Total	5.47	0.17	3.96	6.61	5.10	5.83	

Abbreviations: Max., maximum; Min., minimum; SE, standard error of mean.

**Table 5:** Spore-forming count of milk sample in log10 CFU/mL.

	Sampling points	Mean	SE	Min.	Max.	95% confidence interval for mean		*p* value
						Lower bound	Upper bound	
Sampling points	Udder	1.93	0.86	0.00	3.96	0.70	3.16	< 0.001
	Milk container/bucket	4.89	0.38	3.66	6.48	3.66	6.11	
	Cafeteria	5.57	0.32	4.26	6.36	4.34	6.80	
	Total	4.13	0.49	0.00	6.48	3.42	4.84	

Season	Summer	3.65	0.72	0.00	5.60	2.17	5.14	0.16
	Winter	4.61	0.68	0.00	6.48	3.12	6.09	
	Total	4.13	0.49	0.00	6.48	3.08	5.18	

Districts	Axum	4.51	0.10	0.00	6.48	2.69	6.33	0.37
	Shire	3.13	1.06	0.00	6.36	1.31	4.95	
	Sheraro	4.75	0.29	3.96	5.63	2.93	6.57	
	Total	4.13	0.49	0.00	6.48	3.08	5.18	

Abbreviations: Max., maximum; Min., minimum; SE, standard error of mean.

**Table 6:** Psychrotrophic count at different sampling points in log10 CFU/mL.

	Sampling points	Mean	SE	Min.	Max.	95% confidence interval for mean		*p* value
						Lower bound	Upper bound	
Sampling points	Udder	5.13	0.27	5.77	4.24	4.557	5.70	0.48
	Milk from container/bucket	5.36	0.28	6.18	4.58	4.785	5.93	
	Cafeteria	5.53	0.31	6.53	4.67	4.957	6.10	
	Yogurt	5.72	0.24	6.43	4.83	5.150	6.30	
	Total	5.44	0.14	6.53	4.24	5.149	5.72	

Season	Summer	5.94	0.11	6.53	5.37	5.682	6.20	< 0.001
	Winter	4.93	0.14	5.64	4.24	4.673	5.19	
	Total	5.44	0.14	6.53	4.24	5.253	5.62	

Agroecology	Axum	5.94	0.17	6.53	5.30	5.392	6.29	0.039
	Shire	5.19	0.27	6.31	4.40	4.942	5.84	
	Sheraro	5.07	0.20	5.78	4.24	4.625	5.52	
	Total	5.40	0.14	6.53	4.24	5.176	5.70	

Abbreviations: Max., maximum; Min., minimum; SE, standard error of mean.

**Table 7:** Determination of bacterial load of some selected bacteria.

Types of bacteria	Sampling points	Mean	SE	Min.	Max.	95% confidence interval for mean		*p* value
						Lower bound	Upper bound	
*E. coli*	Udder	3.26	1.06	0.00	5.69	2.03	4.49	0.017
	Farm	5.42	0.23	4.66	5.96	4.19	6.65	
	Cafeteria	5.62	0.39	3.66	6.08	4.39	6.85	
	Yogurt	5.97	0.25	4.77	6.55	4.74	7.20	
	Total	5.07	0.30	0.00	6.55	4.45	5.68	

*Salmonella* species	Udder	2.97	1.00	0.00	6.09	1.81	4.14	< 0.006
	Farm	5.38	0.22	4.74	5.98	4.22	6.54	
	Cafeteria	5.79	0.20	5.02	6.42	4.62	6.95	
	Yogurt	5.58	0.39	4.13	6.51	4.41	6.74	
	Total	4.93	0.35	0.00	6.51	4.35	5.51	

*S. aureus*	Udder	4.65	0.96	0.00	6.46	2.84	6.47	0.04
	Farm	4.97	1.02	0.00	6.59	3.15	6.79	
	Cafeteria	4.97	1.00	0.00	6.30	3.15	6.79	
	Yogurt	5.73	0.30	4.66	6.59	3.91	7.54	
	Total	5.08	0.41	0.00	6.59	2.84	6.47	

Abbreviations: Max., maximum; Min., minimum; SE, standard error of mean.

**Table 8:** Bacterial load of some selected bacteria to determine season and agroecology.

Types of bacteria	Sampling points	Mean	SE	Min.	Max.	95% confidence interval for mean		*p* value
						Lower bound	Upper bound	
*E. coli*	Summer	5.51	0.23	3.96	6.55	4.49	6.53	0.22
	Winter	4.62	0.66	0.00	6.21	3.60	5.65	
	Total	5.07	0.35	0.00	6.55	4.34	5.79	
	Axum	5.62	0.21	4.56	6.08	4.34	6.90	0.42
	Shire	4.46	0.73	0.00	6.55	3.18	5.74	
	Sheraro	5.12	0.74	0.00	6.21	3.84	6.39	
	Total	5.07	0.35	0.00	6.55	4.33	5.80	

*Salmonella* species	Summer	4.45	0.65	0.00	6.51	3.44	5.46	0.18
	Winter	5.41	0.24	3.66	6.30	4.40	6.42	
	Total	4.93	0.35	0.00	6.51	4.21	5.64	
	Axum	5.39	0.32	3.96	6.42	4.10	6.68	0.20
	Shire	4.41	0.69	0.00	6.30	3.12	5.70	
	Sheraro	4.99	0.75	0.00	6.51	3.70	6.28	
	Total	4.93	0.35	0.00	6.51	4.18	5.67	

*S. aureus*	Summer	4.03	0.71	0.00	6.13	2.97	5.09	< 0.008
	Winter	6.13	0.12	5.18	6.59	5.07	7.19	
	Total	5.08	0.41	0.00	6.59	4.33	5.83	
	Axum	5.56	0.21	4.94	6.44	4.17	6.95	0.084
	Sheraro	3.80	1.13	0.00	6.59	2.41	5.19	
	Shire	5.88	0.09	5.46	6.18	4.49	7.27	
	Total	5.08	0.41	0.00	6.59	4.28	5.88	

Abbreviations: Max., maximum; Min., minimum; SE, standard error of mean.

## Data Availability

Data and material are available with the authors and available to other researchers upon request.

## Supporting Information

Supportive information contains photographs of laboratory works.
