# Supplementary material for: Investigation of Microbial Quality of Milk and Milk Products and Isolations of Some Major Bacteria in the Central and Northwestern Zones of Tigray, Ethiopia
Source: Vet Med Int. 2024 Dec 23;2024:9989527. doi: 10.1155/vmi/9989527 (PMC11685325; doi:10.1155/vmi/9989527)
Supplement: Supporting Information — Additional supporting information can be found online in the Supporting Information section. [file 9989527.f1.docx]

**Aksum University Shire Campus**

**Laboratory Work in Department of Animal Science**

**Microbiology Laboratory**


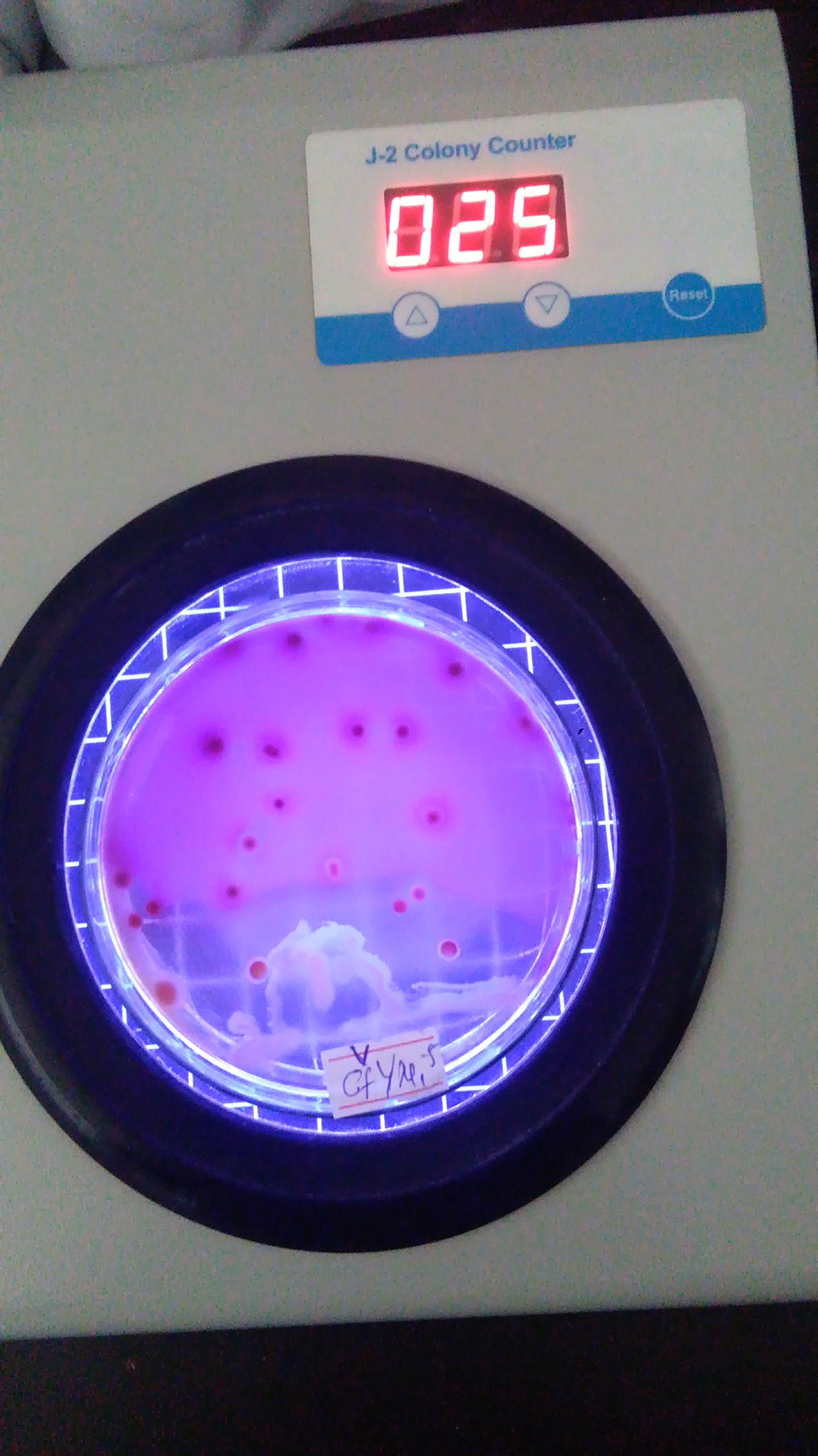


Figure S1: Coliform bacteria colonies were grown on violet red bile agar (VRBA) in serial dilution of 0.00001.


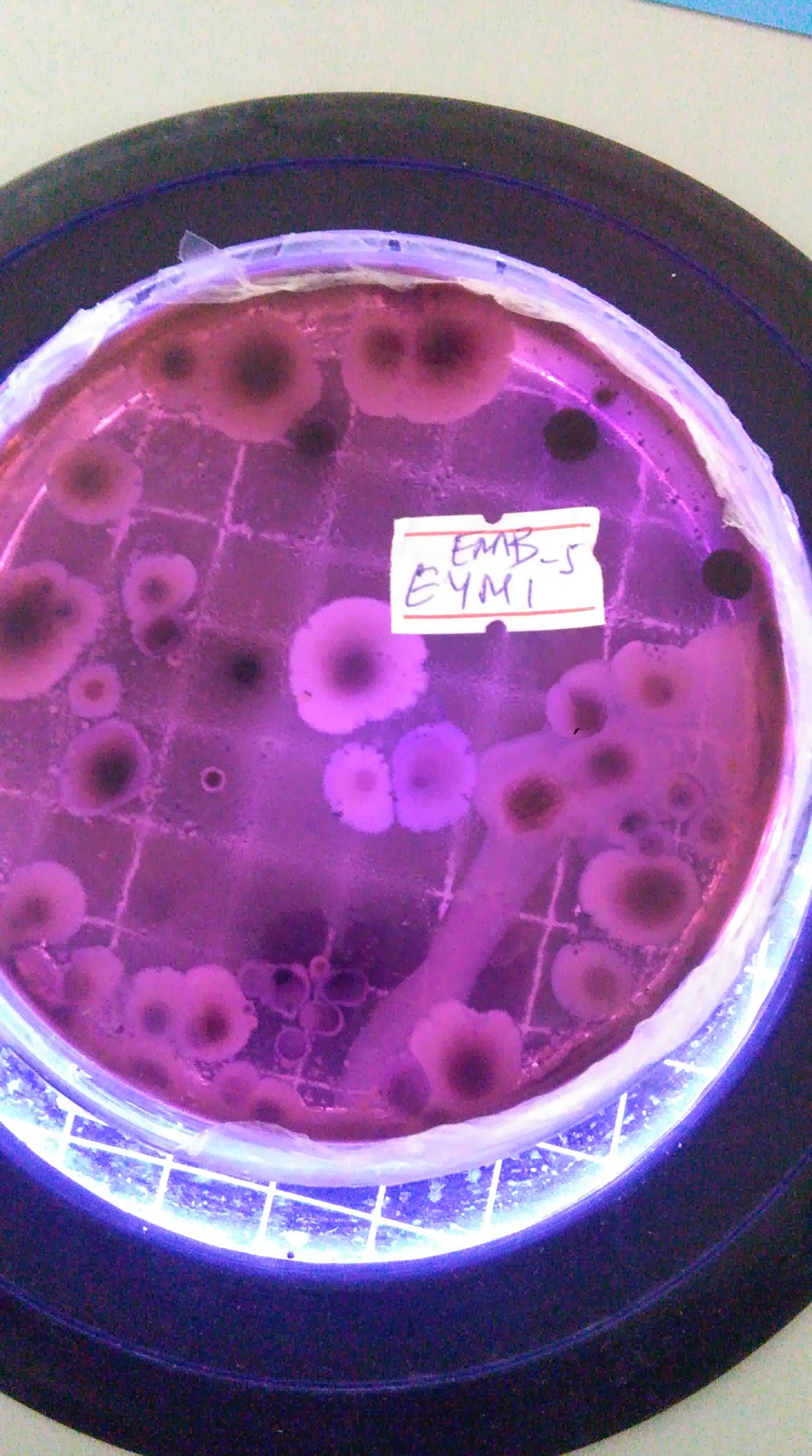


Figure S2: *Escherichia coli* colonies were grown on Eosin Methylene Blue (EMB) agar in serial dilution of 0.00001.


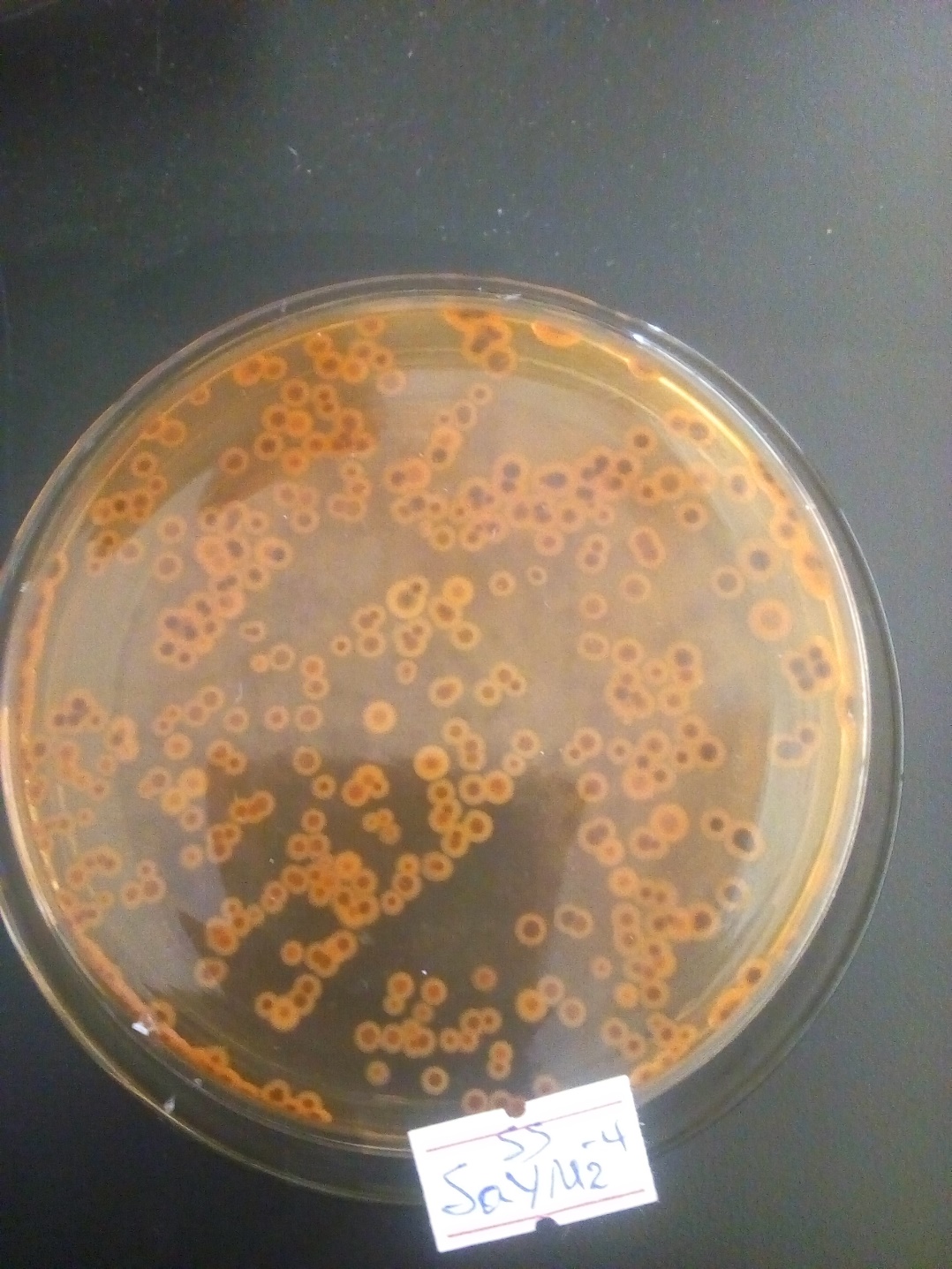


Figure S3: *Salmonella* colonies were grown on Selenite Cystine Broth in a serial dilution of 0.0001


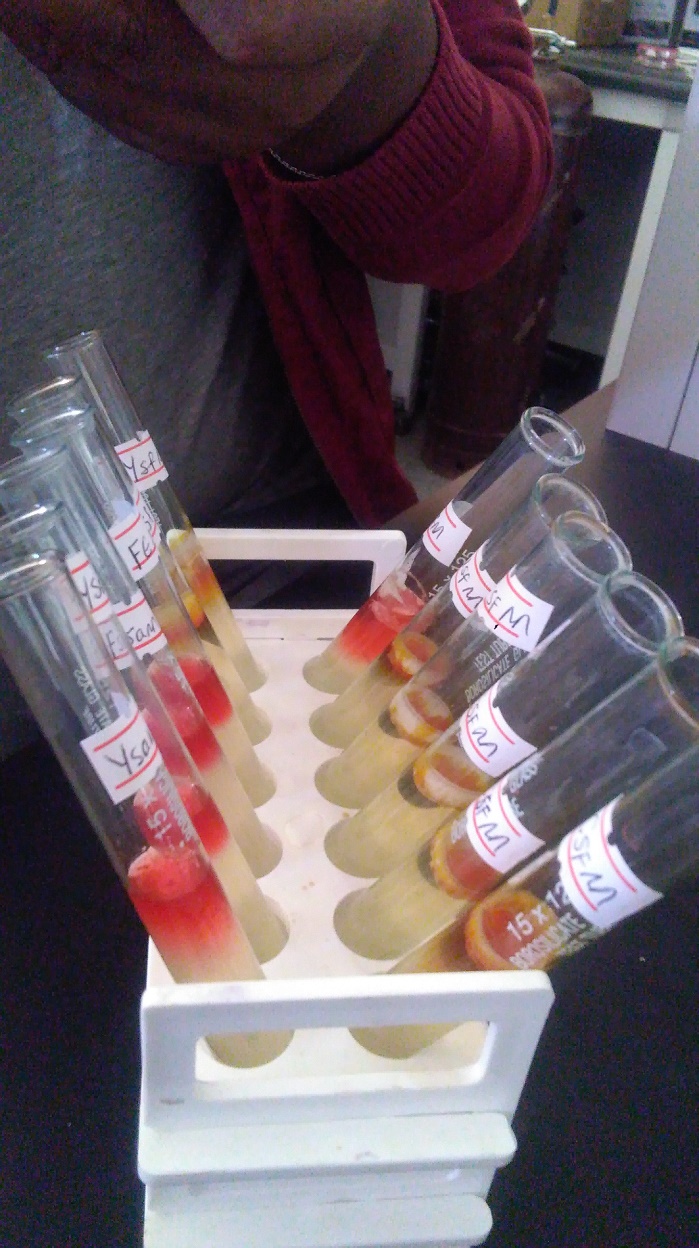

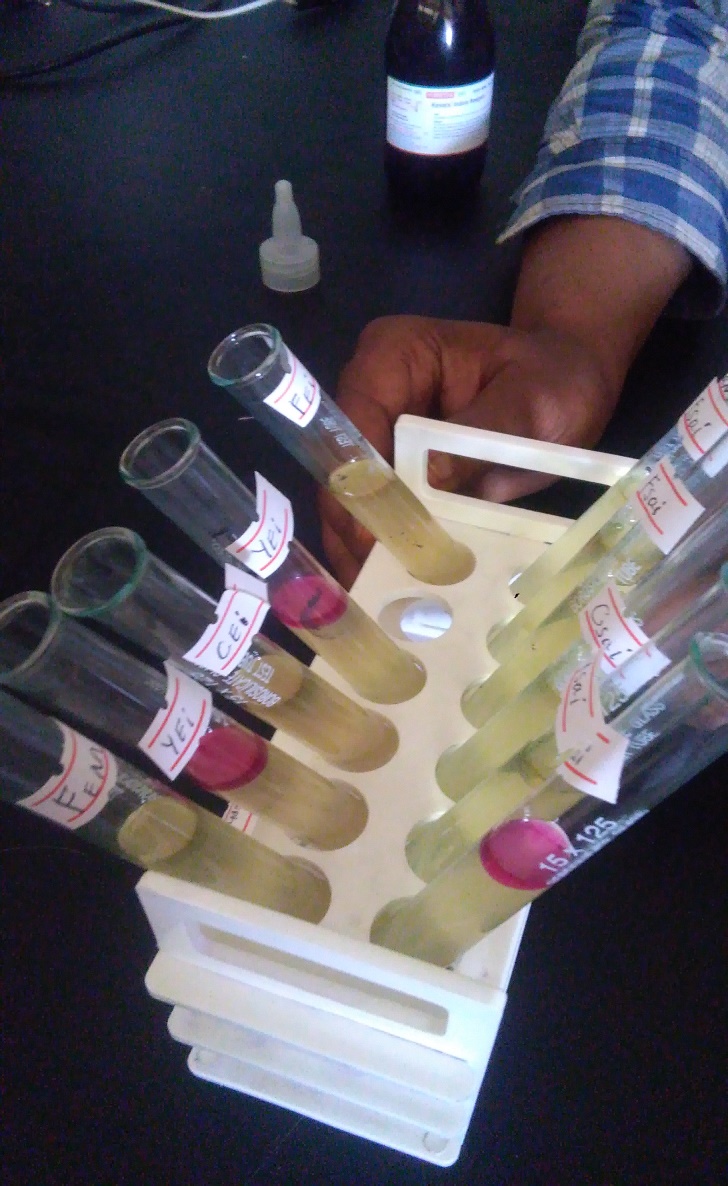


Figure S4: Biochemical tests: methyl red test (lift) and indole test (right) were carried out to confirm *Staphylococcus aureus*, *Escherichia coli* and *Salmonella spp.*
